# Tribocorrosion Behavior of Micro/Nanoscale Surface Coatings

**DOI:** 10.3390/s22249974

**Published:** 2022-12-17

**Authors:** Md Ashraful Hoque, Chun-Wei Yao, Mukunda Khanal, Ian Lian

**Affiliations:** 1 Department of Mechanical Engineering, Lamar University, Beaumont, TX 77710, USA; 2 Department of Biology, Lamar University, Beaumont, TX 77710, USA

**Keywords:** tribocorrosion, wear-corrosion, micro/nano coatings, superhydrophobic coatings

## Abstract

Wear and corrosion are common issues of material degradation and failure in industrial appliances. Wear is a damaging process that can impact surface contacts and, more specifically, can cause the loss and distortion of material from a surface because of the contacting object’s mechanical action via motion. More wear occurs during the process of corrosion, in which oxide particles or debris are released from the contacting material. These types of wear debris and accumulated oxide particles released during corrosion cause a combination of wear-corrosion processes. Bringing together the fields of tribology and corrosion research, tribocorrosion is a field of study which deals with mechanical and electrochemical interactions between bodies in motion. More specifically, it is the study of mechanisms caused by the combined effects of mechanical stress and chemical/electrochemical interactions with the environment. Tribocorrosion testing methods provide new opportunities for studying the electrochemical nature of corrosion combined with mechanical loading to establish a synergistic relationship between corrosion and wear. To improve tribological, mechanical, and anti-corrosion performances, several surface modification techniques are being applied to develop functional coatings with micro/nano features. This review of the literature explores recent and enlightening research into the tribocorrosive properties of micro/nano coatings. It also looks at recent discussions of the most common experimental methods and some newer, promising experimental methods in tribocorrosion to elucidate their applications in the field of micro/nano coatings.

## 1. Introduction

The process of tribocorrosion should be better understood to design new materials for service applications involving the simultaneous action of mechanical and electrochemical agents on engineering components. Tribocorrosion is a synergetic process that results from the combination of mechanical loads and chemical reactions among tribosystem components operating under adverse circumstances. Due to the influence of mechanical and chemical interactions, such a system can undergo a variety of negative changes to its body, the interfacial medium, and the counter body. Because of simultaneous mechanical and chemical events, tribocorrosion cannot be reduced to the simple addition of wear and corrosion. Understanding and controlling tribocorrosion hence demands a distinct strategy.

The mechanistic approach is one of the simplest methods adopted by researchers [1,2,3,4,5]. It determines the combined mechanical and chemical wear volume loss in a tribocorrosion system. Another way to study tribocorrosion is through the synergistic approach [6,7,8,9]. The assumption of a synergy term separates this strategy from the mechanistic approach, but otherwise they are virtually identical. Due to the interaction between mechanical and chemical breakdown, the term “synergy” encompasses both wear-induced corrosion and corrosion-induced wear.

In a tribocorrosion test, the contact area of the wear process is completely submerged in the test electrolyte, allowing for simultaneous wear and corrosion experiments to be conducted using a ball/pin-on-disc [10,11] or sliding ball-on-plate [12,13] configuration. Using nanoindentation [14,15] technology, scientists have developed several cutting-edge methods in recent years for gauging the mechanical properties of coatings. Researchers have been using it to conduct nano-DMA [16] and nano-scratch [17,18,19] tests to measure storage modulus, loss modulus, and abrasion resistance. In addition, frictional properties have been studied with AFM’s lateral force microscopy (LFM) [20,21], as demonstrated by numerous important works of research over the past decade. Open circuit potential (OCP) [22,23], potentiodynamic [24] and potentiostatic polarization [25], and electrochemical impedance spectroscopy (EIS) [26,27] can also be recorded in real-time during a tribocorrosion test by applying a known force to a pin or ball simultaneously in contact with the surface of the specimen to induce wear. Many researchers have investigated surface properties roughness measurement [28,29] and contact angle measurement [30,31]. Observing the morphology of worn areas [32,33] is also crucial for learning about the wear process.

Metals [34,35], their alloys [36,37], and derivative metallic or alloy coatings [38,39,40] are significant materials to be studied in tribocorrosion. High-entropy alloys (HEAs) are a newer class of metals that have been shown to have improved mechanical capabilities at high temperatures and resistance to corrosion [41,42]. HEAs’ superior hardness, thermal stability, wear, oxidation, and corrosion resistance come from the fact that they often consist of simpler phases such as FCC and BCC, owing to their high mixing entropy [43]. Most of the research in this area has focused on determining corrosion properties and mechanical properties along with tribocorrosive properties [44,45,46]. Until now, HEA coatings have been deposited using a variety of techniques including laser cladding [47], magnetron sputtering [48], thermal spraying [49], electrochemical deposition [50], and others. The HEA coatings’ microstructure is obviously different from that of bulk HEAs because of the preparation method’s fast cooling rate, which makes it easier to produce amorphous and nanocrystalline structures and achieve higher mechanical and tribocorrosive properties [51,52].

Due to their excellent resistance to wear and corrosion, ceramic materials are often utilized as protective coatings for marine engineering components in saltwater conditions. The plasma electrolytic oxidation (PEO) process is a low-cost and high-efficiency surface modification method that can be used to prepare ceramic coatings for metal surfaces. PEO is also known as micro arc oxidation (MAO), which can be used to fabricate dense oxide coatings with superior wear and anticorrosion performance over soft metal surfaces [53]. MAO coatings deposited on the surface of soft metal alloys are composed of rutile and anatase phases, giving them excellent tribocorrosive performance [54]. Nano particle reinforcement in a MAO-treated surface also causes a triplex structure (barrier film, inner porous layer, and outer porous layer), which gives it superior tribocorrosive performance [55]. Physical vapor deposition (PVD) technology has been used to prepare metal nitride coatings for a variety of applications, including corrosion resistance, wear resistance, and surface decoration, and these coatings have shown great promise in enhancing the anti-wear and anti-corrosion performances of marine engineering machinery [56,57,58]. Marchin and Ashrafizadeh [59] examined the effectiveness of TiSiCN and TiSiN coatings on cold forming steel dies and studied the impact that carbon addition had on the tribological behavior of a multilayered, nanostructured TiSiN coating. The coatings were applied by a cathodic arc physical vapor deposition (CAPVD) technique used in industry. Friction patterns between the two coatings implied that TiSiCN was more naturally lubricous than the other. This was likely due to the presence of graphitic and amorphous carbon. Additionally, using CAPVD, Çomaklı [60] deposited ceramic coatings of various thicknesses and compositions onto the Ti45Nb implant material, including TiN, TiAlN single layers, and TiAlN/TiN multilayer nanostructured coatings. The multilayered coatings showed improved corrosion resistance and lower wear rates compared to the single-layer coating and untreated Ti45Nb substrate. Diamond-like carbon (DLC) films have been found as a promising candidate for a protective material to improve wear and corrosion behavior due to their high hardness, chemical inertness, low wear rate, and high corrosion resistance [61,62]. DLC thin films are composed of sp2- and sp3-hybridized carbon, which gives them attractive mechanical, tribological, chemical, and biological properties [63,64].

Polymer coatings provide powerful functions to their host materials, whether they are relatively basic coatings or nanoparticle-incorporated, functionalized composite coatings. They have several potential uses, such as corrosion-resistant coatings, surface functionalizers, wear-resistant materials, bioactivity enhancers, and even as switchable smart materials [65]. Polydimethylsiloxane (PDMS), polyurethane (PU) [66], polyvinylpyrrolidone (PVP) [67], perhydropolysilazane (PHPS) [68], and epoxy resin are some potential candidates used in polymer-based coating matrixes due to their good adhesion, mechanical properties, and corrosion resistance. Due to their nontoxicity, polymer materials are a good candidate for biocompatibility.

One of the prominent research topics in corrosion science deals with the fabrication of a superhydrophobic coating with superior anti-corrosion and good mechanical properties. The superhydrophobic coating possesses a significant microstructure, which provides a greater air cushion to trap air and protect the substrate from corrosive media. Superhydrophobic coatings are usually a millimeter to a nanometer thick and suffer crucial abrasion damage over their lifetime. Incorporating nanoparticles into the superhydrophobic coating matrix has become popular practice, as nanoparticles are known to increase adhesion strength [69] and scratch or abrasion resistance [70,71]. This review of the literature found almost no studies to determine the tribocorrosive properties of nanocomposite-embedded, polymer-based superhydrophobic coatings. The few studies that exist are limited to coatings that are hydrophobic [72,73] or hydrophilic in nature only [74,75]. Moreover, synergistic study of wear-corrosion has not been performed to characterize the tribocorrosive behavior of superhydrophobic coatings. By addressing these gaps in the literature, this review invites researchers to explore the aforementioned trends in new experimental methods to broaden and deepen the work in these areas.

## 2. Experimental Procedures for Tribocorrosion Tests

### 2.1. Tribocorrosion Tests under Different Configurations

Standard tribo-testing equipment includes a wide range of tribometers with varying designs. The ball-on-disc, pin-on-disc, ball-on-plate, block-on-ring, and ball-on-three-plates combinations are the most popular tribological testing configurations. For tribocorrosion testing, researchers have extensively used ball/pin-on-disc and ball-on-plate configurations due to their simple principle and versatility. The principle behind both the ball-on-disc and pin-on-disc arrangements is the same; what changes is the nature of the contact between the two. Ball/pin-on-disc tribometers provide universal experimental setups that are often used to conduct tribological research during the prototype phase, prior to full-scale testing. The ball/pin-on-disc tribometer model analyzes friction and wear processes in sliding movement on a circular track. Mechanical and tribocorrosive properties of an Ni-W alloy and Ni-W-BN(h) composite coating on Fe substrate were investigated by Huang et al. [10]. For the tribocorrosion test, they utilized a ball-on-disc abrasion tester and a potentiostat with a 3.5 wt.% NaCl solution at room temperature. Based on tribocorrosion test results, it seems that the BN(h) particles’ self-lubricating characteristics helped lower the coating’s friction coefficient. Using a pin-on-disc tribometer with a 20 N load, Sarrivirta et al. [11] investigated the tribocorrosive behavior of three thermally sprayed cermet coating types in an aqueous environment comprising chlorides and sulfates at a pH value of 4.5. Powder-pack boronizing was used by Aichholz et al. [76] on AISI 4140 steel to create a monophasic iron boride (Fe_2_B) layer. A pin-on-disc tribometer was used to measure tribological behavior in a 3.5% NaCl solution using a potentiostat. In both dry and wet tribological settings (3.5 wt.% NaCl solution), the boronized surface showed significantly reduced wear rate values compared to unboronized specimens.

**Figure 1:**
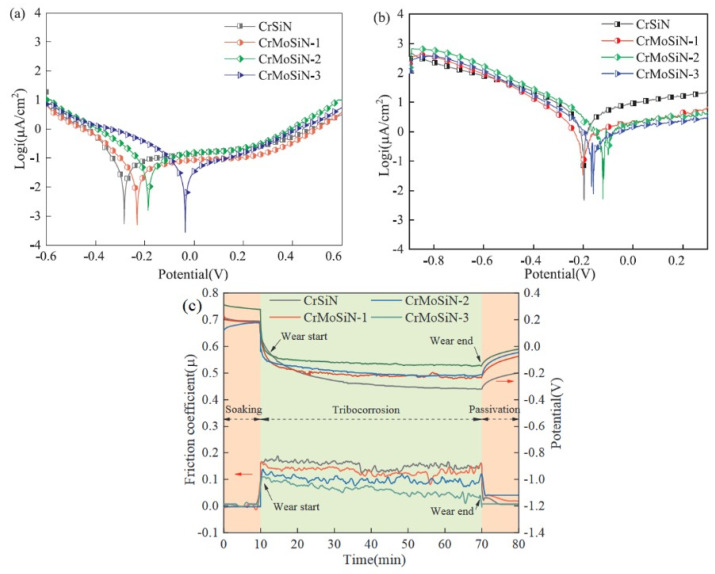
Potentiodynamic polarization test results under (**a**) static corrosion, (**b**) tribocorrosion. (**c**) Tribocorrosion under OCP. (Reprinted from Applied Surface Science, 525, Y. Fu, F. Zhou, M. Zhang, Q. Wang, and Z. Zhou, Structural, mechanical and tribocorrosion performances of CrMoSiN coatings with various Mo contents in artificial seawater, 146629, Copyright (2020), with permission from Elsevier).

The ball in contact with the flat plate travels in a reciprocating motion along a linear route, as opposed to the ball/pin-on-disc setups, in which motion occurs in a single direction over a circular track. With the linear wear option, the tribometer can simulate the linear motion seen in a wide range of real-world systems [77]. Guo et al. [12] used the MAO technique to coat Ti6Al4V alloy in electrolytes containing CNTs. They investigated the impact of CNT content on the corrosion/tribocorrosion performance of the MAO coating in a 3.5 wt.% NaCl solution. The greatest OCP value was seen in the MAO coatings produced in an electrolyte containing 0.15 g/L CNTs, together with the lowest wear rate and COF during sliding. Gnanavelbabu et al. [78] examined the tribocorrosive and electrochemical corrosive behavior of AA2014/Al_2_O_3_ (1–4 wt.%) nanocomposites. Under potentiodynamic polarization conditions, a tribocorrosion test was carried out in a linear reciprocating tribometer utilizing a 3.5 wt.% NaCl solution as the electrolyte. The AA2014/3 wt.% Al_2_O_3_ nanocomposite showed the lowest potential with the lowest current density, according to the findings of the tribocorrosion tests. The corrosion kinetics of MAO-treated Ti surfaces generated in electrolytes with varying amounts of calcium acetate were compared by Sousa et al. [13]. Surfaces treated with MAO showed slower tribocorrosion kinetics and less wear damage compared to untreated Ti. They confirmed that the presence of rutile in the MAO layer mitigated the mechanical damage caused by the tribocorrosive process when calcium acetate was present in higher quantities. CrMoSiN coatings were produced by Fu et al. [79] utilizing unbalanced magnetron sputtering on Si wafers and titanium alloy substrates with varying molybdenum (Mo) percentages. With a linear reciprocating motion of the tribometer, they studied the tribocorrosive behavior of coatings sliding across SiC balls in saltwater. Figure 1a,b represent potentiodynamic polarization curves under static corrosion and sliding contact condition, respectively. The OCP values of coatings made of CrSiN and CrMoSiN sliding against SiC balls are shown in Figure 1c as a function of testing time.

To represent a line contact between the two interacting surfaces, the block-on-ring geometry was used [80]. The block-on-ring setup involves applying a controlled force to a test block while it presses against a test ring. The coefficient of friction between the block and the ring is measured while the ring is rotated at a predetermined speed. Journal bearings, rings, lubrication films, etc. are often tested for COF and wear using tribometers with this sort of setup [80]. Researchers have incorporated this test setup under electrochemical conditions to measure the tribocorrosive properties of micro/nanostructured surfaces [81,82,83,84]. The ball-on-three-plates arrangement is another structure used by a few laboratories [85,86] for estimating lubricant tribological behavior. A spherical ball is paired with a set of three plates that can move in any direction to create this setup. This setup has not been used often in tribocorrosion experiments on coated materials.

### 2.2. Tribocorrosion Tests under Different Electrochemical Conditions

**Figure 2:**
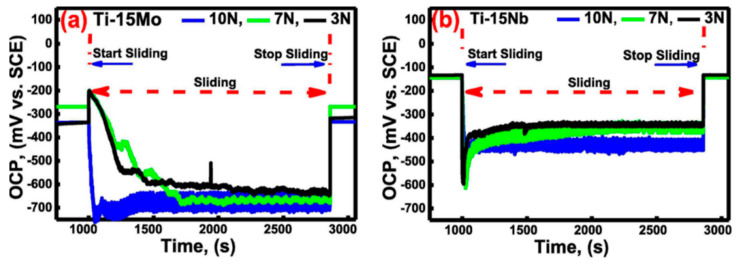
The OCP evolution during tribocorrosion under varying load: (**a**) Ti-15Mo, (**b**) Ti-15Nb. (Reprinted from Materials Letters, 257, M. Fellah, N. Hezil, M. A. Hussein, M. A. Samad, M. Z. Touhami, A. Montagne, A. Iost, A. Obrosov, and S. Weiss, Preliminary investigation on the bio-tribocorrosion behavior of porous nanostructured β-type titanium based biomedical alloys, 126755, Copyright (2019), with permission from Elsevier).

The literature review showed that researchers have adopted different types of tribological testing configurations (most commonly ball/pin-on-disc and ball-on-plate) and combined them with different electrochemical techniques such as open circuit potential (OCP), potentiodynamic polarization (PDP), potentiostatic polarization (PSP), and electrochemical impedance spectroscopy (EIS). Using OCP measurement techniques, one may learn if a metal, for instance, is in an electrochemically active or passive state. During sliding testing, the open circuit potential is a composite potential, representing both the condition of the pristine disc material and the condition of the material in the wear track [22]. Shittu et al. [23] tested the tribocorrosion reaction of a laser-engineered, net-shaped CoCrFeMnNi HEA in a room-temperature, 3.5 wt.% NaCl solution under OCP conditions. During the tribocorrosion test, the additively manufactured alloy showed excellent surface re-passivation as evidenced by the open circuit potential curves, which showed a sharp drop to more negative values as wear began, followed by continuous change for the duration of the active tribocorrosion phase. Cui et al. [87] used a DC reactive magnetron sputtering technique to create a nanocrystalline TiZrN graded layer on a biomedical titanium alloy. Researchers studied the tribocorrosion behavior of the coated titanium alloy in Hank’s solution at open circuit potential (OCP) and applied potentials (−0.15 V to +0.25 V). The TiZrN coating’s stable passive film compositions and great resistance to plastic deformation explain why its OCPs drop by less during friction under OCP conditions. Fellah et al. [88] studied the tribocorrosion behavior of porous nanostructured β-type titanium-based biomedical alloys in phosphate-buffered saline (PBS) under various stresses and OCP conditions. Figure 2a,b indicate that immediately upon sliding start, all specimens exhibited a negative potential shift, with the magnitude being greater for Ti-15Mo than for Ti-15Nb. Both alloys showed a positive potential shift towards the end of the sliding process, and the OCP quickly rose to its initial values. The passive layer on the wear track of the samples was re-passivated, restoring the samples’ noble behavior by creating a barrier against corrosion.

**Figure 3:**
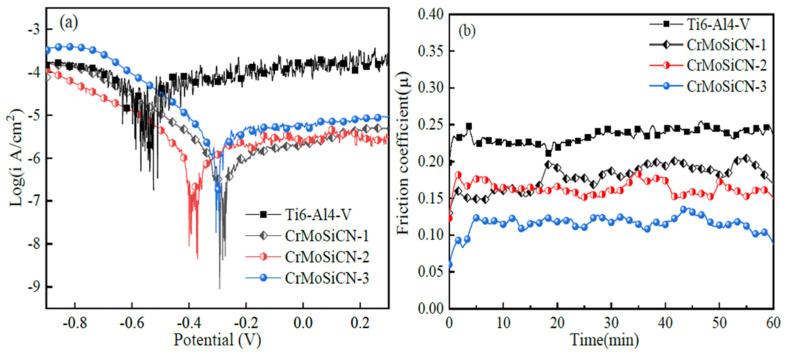
The effects of potentiodynamic polarization on the tribocorrosion behaviors of Ti6Al4V and CrMoSiCN coatings as they slide over SiC balls: (**a**) the polarization curves and (**b**) the friction coefficient variation. (Reprinted from Corrosion Science, 165, Y. Fu, F. Zhou, Q. Wang, M. Zhang, and Z. Zhou, Electrochemical and tribocorrosion performances of CrMoSiCN coating on Ti-6Al-4V titanium alloy in artificial seawater, 108385, Copyright (2020), with permission from Elsevier).

Potentiodynamic polarization during tribocorrosion tests has allowed researchers to observe the effects of friction on different electrochemical reactions taking place, resulting in a wide range of applied potentials [89]. This method is useful in determining the active/passive behavior of materials at different potentials. Da et al. [24] optimized the interfacial adhesive between GO and CBPCC by coating its surface with nano ZnO (GO-ZnO). As a further measure, GO-ZnO was used as reinforcement in CBPCC to make them more resistant to tribocorrosion. Potentiodynamic polarization results during sliding revealed that the corrosion resistance of CBPCC was shown to improve when GO-ZnO content was increased. Shivaram et al. [90] used powder metallurgy to create a new Ti-20Nb-5Ag alloy with a porous, micro/nano structured surface. The corrosive tribological behavior of the porous alloy was studied using PDP in simulated bodily fluid at loads ranging from 1 to 10 N. Results showed that corrosion current density and corrosion rate significantly increased with higher applied loads, owing to passive film breakdown. With a closed-field unbalanced magnetron sputtering technique, Fu et al. [91] created CrMoSiCN coatings with varying Mo concentrations. Different CrMoSiCN coatings with varying Mo concentrations were tested for tribocorrosion by sliding them against SiC balls in a potentiodynamic environment. The CrMoSiCN coatings with varying Mo concentrations were shown to exhibit potentiodynamic polarization curves under sliding conditions, as seen in Figure 3a. It was discovered that the Ti6Al4V substrate exhibited pronounced fluctuations in the current density of anodic polarization curves, whereas the CrMoSiCN coatings showed a more subtle fluctuation pattern. The friction coefficient of CrMoSiCN coatings sliding against SiC balls under polarization conditions in artificial seawater is shown in Figure 3b. The friction coefficient of CrMoSiCN coatings clearly varied over a narrow range and decreased with increasing Mo content.

**Figure 4:**
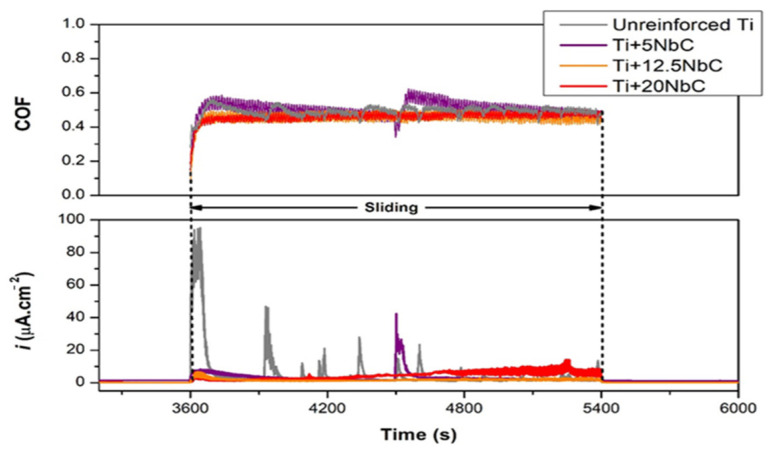
Changes in coefficient of friction and current density during anodic tribocorrosion tests. (Reprinted from Metals 2022, 12(6), V. R. M. Gonçalves, I. Çaha, A. C. Alves, F. Toptan, and L. A. Rocha, Improved Tribocorrosion Behavior Obtained by In-Situ Precipitation of Ti2C in Ti-Nb Alloy, 908, Copyright (2022) by the authors. Licensee MDPI, Basel, Switzerland. This article is an open access article distributed under the terms and conditions of the Creative Commons Attribution (CC BY) license (https://creativecommons.org/licenses/by/4.0/) (accessed on 23 November 2022)).

The tribometer with three-electrode setup has allowed the application of a constant potential (potentiostatic condition) to the interface, with the resulting tribocorrosion current serving as a measurable indicator of the electrochemical reactions occurring there. Goncalves [25] created innovative in situ Ti-based matrix composites (TMCs) via reactive hot pressing of Ti + NbC powder blends, and their tribocorrosion behavior was studied in phosphate-buffered solution (PBS). Tribocorrosion tests were performed by a ball on a plate tribometer at open circuit potential (OCP) and in an anodic potentiostatic state. Figure 4 shows that oxide films formed on surfaces ensured that current density values were constant prior to sliding. Unreinforced Ti showed a dramatic increase in current density as sliding began, while composites showed smaller increases in current density, which tended to be more stable. After removing the counter-material, the anodic current density returned to values close to those recorded before sliding, indicating re-passivation of the worn area.

**Figure 5:**
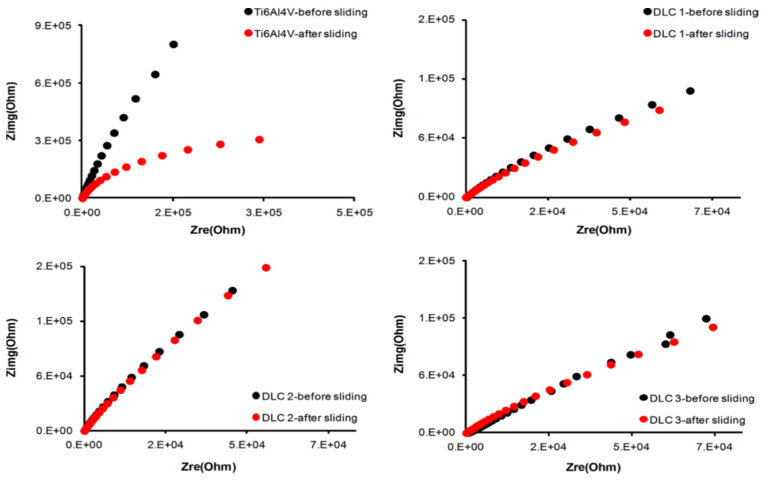
Electrochemical impedance spectra before and after sliding wear test. (Reprinted from Tribology International, 188, R. Bayón, A. Igartua, J. J. González, and U. Ruiz De Gopegui, Influence of the carbon content on the corrosion and tribocorrosion performance of Ti-DLC coatings for biomedical alloys, 115–125, Copyright (2015), with permission from Elsevier).

Gao et al. [26] investigated the electrochemical and tribological properties of the YSZ coating in a 3.5 wt.% NaCl solution after applying it to a 304 stainless steel substrate using atmospheric plasma spraying technology. Tribocorrosion tests were conducted under the action of cathodic to anodic potential and electrochemical impedance spectroscopy (EIS). From the results of comparing the impedance before and after rubbing, the YSZ coating’s impedance performance suffered a slight loss at the cathode potential but showed almost no change at the anode potential. No matter how great 304 stainless steel’s potential, its impedance performance has been found to suffer after being subjected to friction. Zhu et al. [27] aimed to increase pure Ti’s resistance to tribocorrosion by coating TA3 pure titanium with niobium using double-glow plasma alloying. Different samples’ electrochemical impedance spectra were taken before and after being subjected to tribocorrosion. Impedance spectra revealed that the niobium-coated Ti sample showed a larger capacitive loop after sliding compared to its initial state, and this was a good indication of better tribocorrosion resistance. Bayon et al. [92] also performed an electrochemical impedance spectroscopy (EIS) test before and after a sliding wear test on Ti6Al4V alloy and three DLC coatings. Nyquist diagrams (Figure 5) were used to illustrate the changes in the surface’s electrochemical state caused by wear. It was observed that for all DLC coatings, the capacitive loop was almost the same before and after a sliding test, while the Ti6Al4V alloy showed a decrease in the capacitive loop, indicating poor tribocorrosion performance compared to the DLC-coated alloy substrate.

### 2.3. Nanoindentation

**Figure 6:**
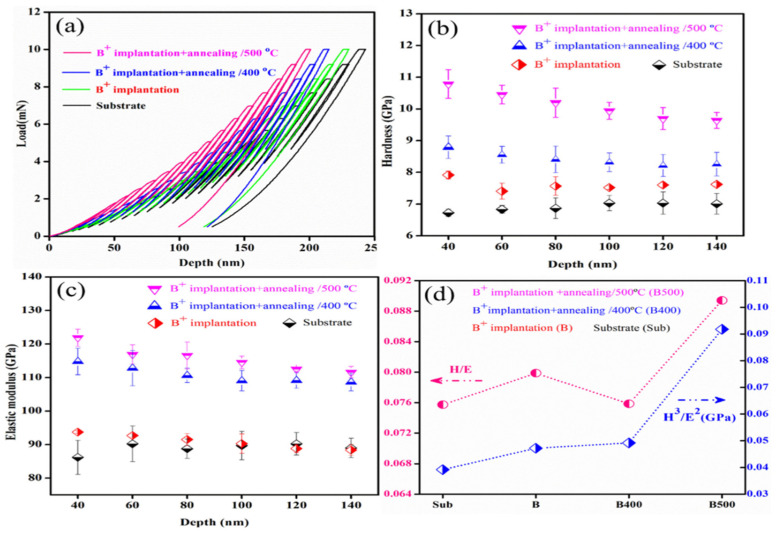
Mechanical properties of the untreated and as-treated 60NiTi surface: (**a**) load–displacement curves; (**b**) hardness (H); (**c**) elastic modulus (E); (**d**) H/E and H^3^/E^2^. (Reprinted from Tribology International, 155, C. Yan, Q. Zeng, W. He, and J. Zhu, Enhanced surface hardness and tribocorrosion performance of 60NiTi by boron ion implantation and post-annealing, 106816, Copyright (2021), with permission from Elsevier).

Nanoindentation is the newest tribocorrosion technique that allows researchers to assess mechanical characteristics such as modulus and hardness of materials in various forms, sizes, and scales [93,94]. This method does not require any pretreatment of the sample, making it the quickest methodology for measuring the characteristics of materials ranging from hard superalloys to soft biomaterials. In recent years, several research groups [14,15,95,96,97] have used the nanoindentation technique in tribocorrosion research to determine hardness and elastic modulus properties of micro/nano structured surfaces. Meghwal et al. [14] conducted an intensive nano and microscale analysis of the plasma-sprayed AlCoCrFeNi coating to correlate the micro/nano structure and mechanical characteristics. The mechanical characteristics of various phases generated during plasma spraying of AlCoCrFeNi HEA have been extensively studied. Statistical analysis of the observed nanoindentation datasets revealed that the black phase of the coating had the maximum hardness, whereas white had the lowest. In addition, the white phase’s pile-up surrounding the alloy phase revealed considerable localized plastic deformation. The same research group used high-velocity oxygen fuel (HVOF) to create an AlCoCrFeNi high-entropy alloy (HEA) micro/nano layer [15]. A major goal of the research was to develop a link between nano- and micro-scale microstructures and mechanical characteristics to better understand the coating performance in high-performance engineering applications. Nanomechanical characteristics of enamel were studied by Xiao et al. [95] to learn how enamel microstructure affects corrosion and tribocorrosion behaviors in both bovine and human enamel. A nanoindenter equipped with a Berkovich indenter was used to assess the hardness and elastic modulus of the enamel surface. For all sample types, the results verified that hardness and elastic modulus visibly declined with increasing corrosion time for normal loads below 100 mN. It has been reported that surface morphologies and roughness can be altered via ion implantation to create micro/nano structured surfaces [98,99]. The high-entropy alloy Al0.07Co1.26Cr1.80Fe1.42Mn1.35Ni1.10 was successfully boronized by nano-sized boronizing powders, as demonstrated by the work of Karakas et al. [96]. Iron borides are created when boron atoms react with steel’s Fe atoms, resulting in the growth of a micro/nano layered surface of iron borides. Nanoindentation measurements were used to learn about the boride layers’ mechanical characteristics. Additionally, wear experiments using a ball-on-a-disc in air, 3.5% NaCl, and 5% H_2_SO_4_ were employed to assess the layers’ tribological performances. The boronized samples achieved surface hardnesses approximately ten times that of the as-cast HEA, as measured by nanoindentation, and the borides were also effective at lowering friction and wear, as evaluated by the tribocorrosion test. To improve the mechanical characteristics and tribocorrosion resistance of 60NiTi when it is exposed to a marine environment, boron ions were implanted into the solution-treated material by Yan et al. [97]. Nanoindentation was used to study the mechanical properties of the near surface of 60NiTi before and after the treatments. The typical indentation load–depth curves of several samples are shown in Figure 6a. These curves were utilized to determine the hardness and elastic modulus variations between the samples. From Figure 6b,c, it can be observed that hardness and modulus were expressed as a function of penetration depth. The boron ion-implanted 60NiTi was clearly superior to the un-implanted sample in terms of hardness and elastic modulus. Figure 6d shows that enhancement in hardness and modulus also caused increased H/E and H^3^/E^2^ ratios.

### 2.4. Dynamic Mechanical Analysis (DMA)

Measurements of properties such as storage modulus, loss modulus, and damping capability (Tan δ) rely heavily on dynamic mechanical analysis (DMA). The modulus can be determined by applying a sinusoidal stress to the material and then measuring the resulting strain. Dynamic mechanical analysis (DMA) is used to characterize the mechanical responses of a material by tracking the variations in its dynamic properties as a function of frequency, temperature, or time. So far, the DMA has been used by a number of researchers as a supporting test to recover the viscoelastic properties of nanomaterials used in the fabrication of micro/nano structured surfaces, alongside the tribological performance. Huang et al. [100] used the sol–gel technique to coat the surface of 304L stainless steel with an epoxy resin (EP) composite coating that was improved with hexagonal boron nitride (HBN) nano-sheets, TiO_2_ particles, and an HBN-TiO_2_ hybrid material. The tribological performance of the polymer was measured with a linear reciprocating tribometer, and its dynamic mechanical behaviors with temperature changes were characterized with a dynamic mechanical analyzer. At lower temperatures, it was discovered that the storage modulus of the composite coatings was very similar to that of pure epoxy. The storage modulus decreased sharply with increasing temperature. The storage modulus decreased most slowly for the HBN-TiO_2_/EP coating. The coefficient of friction and wear rate were found to be lowest when compared to those of pure epoxy resin, proving that the addition of a two-dimensional material (HBN) was successful in reducing friction through interlayer slippage. Di Maro et al. [101] carried out a thorough investigation of how the addition of alumina-toughened zirconia (ATZ) nanoparticles to high density polyethylene (HDPE) affected the composites’ mechanical and tribological performance. Melt extrusion was used to create composites with varying ATZ concentrations in HDPE. The SEM results showed that the insertion of fillers, especially at high percentages, improved the crystallinity of the polymer. Under lubricated conditions, the filler had a favorable impact on the composites’ wear resistance. According to the DMA test, void, agglomeration development, and a rise in crystallinity all appear to be key factors in determining how stiff a composite is. More specifically, composites with little voids and aggregates, or low ATZ amounts, as well as little crystallinity enhancement in the polymer, result in improved polymer stiffness in terms of both Young and storage moduli.

**Figure 7:**
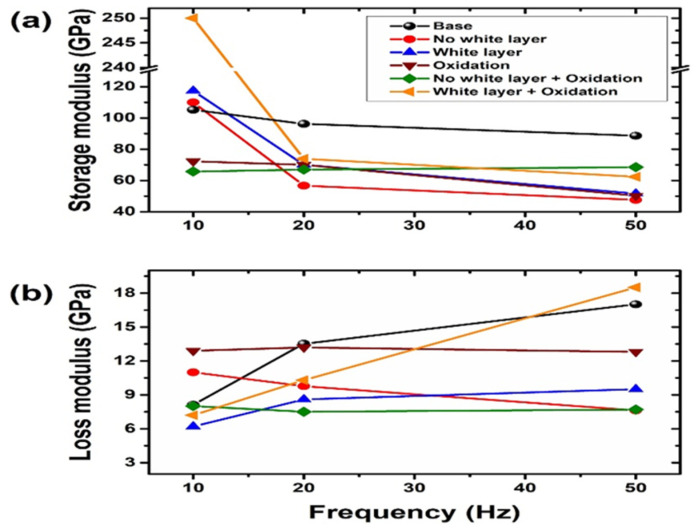
(**a**) Storage modulus and (**b**) loss modulus as a function of cyclic loads of 10, 20 and 50 Hz. (Reprinted from Surface & Coating Technology, 385, J.C. Díaz-Guillén, M. Naeem, H.M. Hdz-García, J.L. Acevedo-Davila, M.R. Díaz-Guillén, M.A. Khan, Javed Iqbal, and A.I. Mtz-Enriquez, Duplex plasma treatment of AISI D2 tool steel by combining plasma nitriding (with and without white layer) and post-oxidation, 125420, Copyright (2020), with permission from Elsevier).

Using a nanoindenter equipped with a dynamic mechanical testing package and a heating/cooling stage, scientists may conduct nanoscale dynamic mechanical analysis (nano-DMA). Nano-DMA is a potential testing method that could be employed in tribocorrosion research to determine the storage modulus, loss modulus, tangent delta, and other properties as a function of frequency, temperature, and contact depth. The mechanical and tribological behavior of a Cu–Zr based metallic glass thin film was examined by Ma et al. [16] for the effect of nano-scale elastic heterogeneity. Using nano-scratch, nano-DMA, and nanoindentation experiments, they studied the room-temperature inhomogeneous plastic deformation and nanotribological characteristics of Cu–Zr based metal glass thin films (MGTFs) produced by magnetron sputtering at varied substrate temperatures. The MGTF elastic heterogeneity was shown directly by nano-DMA experiments, confirming the hypothesis that MGs are fundamentally heterogeneous and include softening areas scattered in an amorphous elastic matrix. Rath et al. [102] used hydroxyapatite (HAp) nanoparticle reinforcement and tripolyphosphate as a cross linker to enhance the mechanical and tribological characteristics of chitosan (CS) films. Cross-linked and uncross-linked CS films with and without HAp reinforcement underwent nano-DMA experimentation. The dynamic loading used in nano-DMA quantifies the time-dependent viscoelastic behavior, including the storage modulus, loss modulus, and tan(δ). Chitosan films containing hydroxyapatite reinforcement and cross-linking demonstrated viscoelastic behavior in the DMA test. Nano-DMA investigations also revealed that both reinforced and cross-linked chitosan films relaxed more quickly. Only HAp-reinforced CS films were subjected to nano scratch tests in order to study nano-tribological behavior. A nano-scratch experiment for chitosan film with hydroxyapatite did not show any substantial pile-up, indicating less plastic deformation. A well-known and effective method for enhancing the surface micro/nano characteristics of many materials, particularly tool and die steels, is plasma nitriding. A white layer is created during high-temperature plasma nitriding, which is bad for tribological performance. No white coating forms while the temperature is low. In order to enhance the AISI D2 tool steel samples’ mechanical and tribological performance, Dáz-Guillén et al. [103] recommended plasma nitriding them at high and low temperatures, generating white layers and no layers, respectively. At room temperature (25 °C), tests for nano dynamic mechanical analysis (nano-DMA) were carried out with a Berkovich tip. The range of frequencies was defined as 10, 20, and 50 Hz. Before the probe oscillated, the indenter was held at its maximum indentation for two minutes. The fluctuations of the storage and loss moduli for the modified surfaces as a function of the loading frequency are shown in Figure 7a,b. At 10 Hz, the white layer both with and without oxidation had a higher loss modulus and a lower storage modulus.

### 2.5. Micro/Nano-Scratch Test

Scratches, or abrasion, are the most common form of damage that occurs to a material in everyday life and leads to degradation in the future. Tests for the coefficient of friction (COF) are often performed to measure the frictional properties of many materials, including lubricants, films, and household objects. Micro-scratch testing is one of the most significant approaches to determine frictional and wear properties of the micro/nano structured surface. This approach has been used by many tribocorrosion research groups [104,105,106,107,108,109,110,111,112]. Torkashvand et al. [113] investigated the mechanical properties, tribological performance, and corrosion resistance of four high-velocity air-fuel (HVAF) sprayed tungsten carbide WC-based coatings with different binders. The tests were conducted in an aqueous solution containing 3.5 wt.% NaCl. The wear and corrosion behaviors of normal WC-CoCr coatings were used as a benchmark for comparison with the behaviors of WC-NiMoCrFeCo, WC-FeNiCrMoCu, and WC-FeCrAl HVAF-sprayed coatings. When compared to the other compositions, the WC-NiMoCrFeCo coating had the best sliding wear performance. In the ball-on-disc test, this coating had the lowest coefficient of friction (CoF) as well as the lowest specific wear rate. In an aqueous solution containing 3.5 wt.% NaCl, the WC-NiMoCrFeCo coatings exhibited superior resistance to corrosion in comparison to the other coatings due to the highly dense micro/nano structure of the coating. Medium-size carbides and a highly corrosion-resistant NiMoCrFeCo matrix could be the reason for excellent dry-sliding behavior and corrosion resistance. By utilizing closed-field, unbalanced magnetron-sputtering ion plating (CFUMSIP), Akhter et al. [114] were able to produce nano structured NiCrN coatings on AISI M2 tool steel substrates. These coatings had varied amounts of Ni. The tribological behavior of these coatings was analyzed by performing reciprocating wear experiments with a pin-on-disc assembly in a room temperature environment. SEM imaging of the wear track confirmed that the failure mode under progressive scratch loading was controlled by the hierarchical nano-structured design of the NiCrN coatings. This design enhanced adhesion and prevented crack propagation through the coating. SEM imaging was performed using a scanning electron microscope. Hoque et al. [105] developed a nanocomposite superhydrophobic top coating on A653 hot-dipped galvanized steel substrate using a spray method. They used a ball-on-plate tribometer in linear reciprocating configuration to determine the static friction coefficient of the superhydrophobic coating to analyze the severity of the corrosion and effectiveness of the coating against corrosion at micro scale. Variation in the COF over 8 mm sliding distance was observed for galvanized steel substrate and superhydrophobic-coated steel substrate. Results indicated that under 1 N and 3 N normal abrasive loads, the superhydrophobic top-coated substrate showed the same coefficient of friction over the sliding distance before and after 90 min of accelerated corrosion, whereas under the same loading condition, galvanized steel substrate showed a much higher COF after accelerated corrosion.

Nano-scratch testing is becoming a well-established nanomechanical characterization method for assessing the mechanical failure behavior and adhesion strength of ceramic coatings and as a modeling tool of single asperity contact in tribological investigations [17,18]. Testing for interfacial adhesion of thin film/substrate systems by scratching with a nanoscale probe has become generally recognized. A reliable indicator of interfacial adhesion strength is the critical load of adhesion failure [19,115]. Identifying the critical load where initial irreversible cracks or fractures are created is the most important part of this procedure. This marks the shift from plastic deformation to significant and lasting damage. The greater the critical load, the stronger the interfacial adhesion [115]. Zheng et al. [116] used a hydrothermal technique to create an Mg (OH)_2_/polypropylene composite coating on the AZ31 Mg alloy, which was then modified with a polypropylene coating. A nanoindentation machine equipped with a Rockwell diamond probe was used to measure the coating’s binding strength. High bonding strength and a high critical load of failure were found in the Mg (OH)_2_/polypropylene coating. Lei et al. [117] adopted a straightforward, solventless, and surfactant-free method to create hydrophobic graphite fluoride (GrF)-reinforced epoxy coatings on steel substrates. The nano-scratch test was performed using a diamond Berkovich tip at a constant scratch velocity of 30 m/s and a scratch distance of 500 m. The scratch load was increased linearly from 0 to 5 mN. The effective scratch penetration depth during scratching and the residual scratch depth after scratching were measured and used to measure the elastic recovery percentage. According to the findings of the investigation of elastic recovery percentage results, elastic deformation and residual depth were reduced by 43% when 1% GrF was added to the epoxy. Samani et al. [118] used NiP and NiP-SiC nanocomposite interlayers to develop diamond-like carbon (DLC) coatings on carbon steel using the RF-PECVD process. As a way to determine how well NiP and NiP nanocomposite interlayers affect the tribological behavior of DLC films, researchers used nano-scratch and nano-indentation to examine how these interlayers affect DLC film structure, adhesion strength, hardness, and friction coefficient. According to critical load studies, adhesion was greatly improved by using NiP and NiP-SiC nanocomposite interlayers. A low friction coefficient of less than 0.05 was reported in DLC films with a NiP-SiC nanocomposite interlayer. Nano-scratch is also a helpful tool for examining the frictional properties (i.e., coefficient of friction) of thin films and coatings under very small loads or high contact pressures at micro or nanoscale. The nanoscale friction coefficient could be very useful to understand the corrosion behavior of a bare metal substrate and superhydrophobic-coated substrate at micro and nano levels [105]. Hoque et al. [105] performed a nano-scratch using a standard Berkovich diamond tip on a zinc-coated steel substrate and superhydrophobic top-coated steel substrate over a sliding distance of 10 μm. For the zinc-coated substrate, a significant increase in the COF after corrosion was observed, while the superhydrophobic top-coated surface retained its COF even after accelerated corrosion. Both micro- and nano-scratch tests confirmed the superior abrasion resistance and anti-corrosion properties of the superhydrophobic top coating.

### 2.6. Lateral Force Microscopy Using AFM

**Figure 8:**
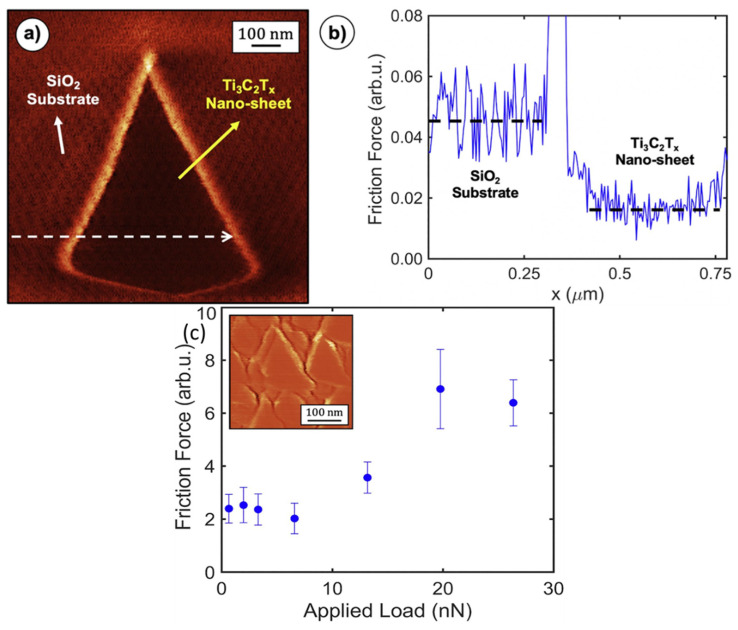
(**a**) Friction map captured on few thin Ti_3_C_2_Tx nanosheet resting on a SiO_2_ substrate. (**b**) Line profile taken from friction map of (**a**) following the white arrow’s dashed line. (**c**) Mean friction calculated as a function of the applied load and measured on several layers of Ti_3_C_2_Tx nano-sheet-based film. (Reprinted from Applied Surface Science, 535, A. Rodriguez, M.S. Jaman, O. Acikgoz, B. Wang, J. Yu, P. G. Grützmacher, A. Rosenkranz, and M.Z. Baykara, The potential of Ti_3_C_2_TX nano-sheets (MXenes) for nanoscale solid lubrication revealed by friction force microscopy, 147664, Copyright (2021), with permission from Elsevier).

One use of AFM, known as lateral force microscopy (LFM), involves the measurement of a force component that is perpendicular to the normal of the surface being examined. Nanoscale friction forces are typically measured using lateral force microscopy (or friction force microscopy). Nanoscale dissipative forces can be measured with a contact-mode sliding tip using LFM/FFM [32]. It is also possible to measure frictional forces at the micro and nanoscale by performing nanoindentation followed by unidirectional sliding (NUS) [119]. Friction forces were analyzed using NUS and LFM by Echeverrigaray et al. [119] on a-C:D/H and a-C:H thin films with varying [D]/[C] and [H]/[C] ratios. A nano-tribometer with a conical diamond tip was used to conduct the NUS tests. Thin films of a-C:D/H and a-C:H were subjected to friction tests, with the tests conducted in a one-way sliding mode. Both nano- and micro-scale measurements of friction forces (using NUS and LFM, respectively) provide insight into the same underlying physical phenomenon, namely, a damping mechanism that is independent of the thickness of the outermost nanolayer. Ni-C nanocomposite thin films’ magnetic domain structure and surface mechanical properties changed with annealing temperature, as shown by Pandey and Kar [21]. Lateral force microscopy was used to conduct an in-depth qualitative analysis of the impact of annealing temperature on the surface friction of the thin films (LFM). Thermal annealing shows potential as a post-deposition process for altering the magnetic domain structure of Ni-C nanocomposite thin films, as evidenced by a decreasing trend in surface friction with increase in annealing temperature. Using atomic force microscopy (AFM) in lateral force microscopy mode, Wood et al. [120] investigated contact friction between orthopedic implant surfaces and the host tissues. Authors simulated the contact behavior between smooth control Ti6Al4V alloy and HTE surfaces against a durable SiO_2_ sphere. At high (5 Hz) and low (0.5 Hz) scan velocities, the friction was assessed in both liquid and air conditions. Friction force decreased in the air and increased in PBS at scan velocities below 2 Hz, stabilizing at scan velocities over 2 Hz. In contrast to surface roughness and nanostructure morphology in air, this may be primarily due to wettability and liquid film layers on the surface. Elam et al. [121] delved into how corrosion affected the friction response and nanoscale topography of a hydrogenated amorphous carbon film (a-C:H). Lateral force microscopy (LFM) was employed ex situ both inside and outside the corroded portions of the coating to evaluate the impact of corrosion on the nanoscale friction response of the a-C:H film. The friction coefficient rose as a function of the degree of corrosion, with the a-C:H film corroded at 2.5 V having the maximum friction coefficient. Authors identified that alterations to the surface topography and surface chemistry both played a role in the observed result. To demonstrate the frictional properties of MXenes on the nanoscale, Rodriguez et al. [122] displayed a friction map obtained by using friction force microscopy on a single Ti_3_C_2_Tx nano-sheet deposited on a silicon dioxide substrate. According to friction maps, few-layer Ti_3_C_2_TX nano-sheets function as solid lubricants on SiO_2_ substrates, reducing sliding friction to a degree that is lower than that of graphene and MoS_2_. As shown in Figure 8a, a friction map was taken on an isolated Ti_3_C_2_Tx nano-sheet with a triangular morphology and a height of roughly 6.5 nm. The friction map line profile is shown in Figure 8b. Measurements of mean friction as a function of applied load were taken on a film consisting of multiple Ti_3_C_2_Tx nano-sheets, and the results are displayed in Figure 8c. The standard deviation is represented by error bars, which were calculated by subdividing the corresponding friction map (inset) into ten regions and discarding the regions that contained nano-sheet edges.

### 2.7. Surface Roughness, Morphology, and Wettability Tests

Asperity interlocking or deformation may enhance friction, hence surface roughness is a crucial element in determining a surface’s tribological behavior [123]. The macroscopic contact angle measurement on the flat surface of any solid substance is crucial to several processes, including moistening, spreading, and wetting, and is strongly affected by the surface roughness of that material [28]. In tribocorrosion research, surface roughness measurement is a very useful method to predict surface behavior after any surface treatment. Visentin [29] used the LP-MOCVD method to produce conformal crystalline TiO_2_ on Ti surfaces with the intention of enhancing the titanium’s surface characteristics. They used a stylus profilometer to measure the surface roughness before and after the MOCVD coating procedures. By using a profilometer, the researchers showed that MOCVD provides excellent conformal coverage on Ti substrates. Siddaiah et al. [124] used a 3D optical profilometer to examine the change in AZ31B surface roughness (SR) brought upon with laser shock peening (LSP). Mean roughness (Sa), maximum peak height (Sp), maximum valley depth (Sv), maximum height (Sz), and change in surface area after LSP were all measured. It was discovered that the SR parameters are linearly affected by the laser intensities used for LSP, with an increase in SR occurring as laser intensity rises. Sebastian et al. [125] looked at the role poly (dimethylsiloxane) (PDMS) played in a silica nanoparticle and toluene-based nanocomposite coating solution. In contact mode, an atomic force microscope was used to assess the roughness of the materials’ surfaces. Height distributions for all coatings were found to be substantially less skewed and, hence, very symmetric, as seen by the surface skewness and kurtosis values from AFM topography.

**Figure 9:**
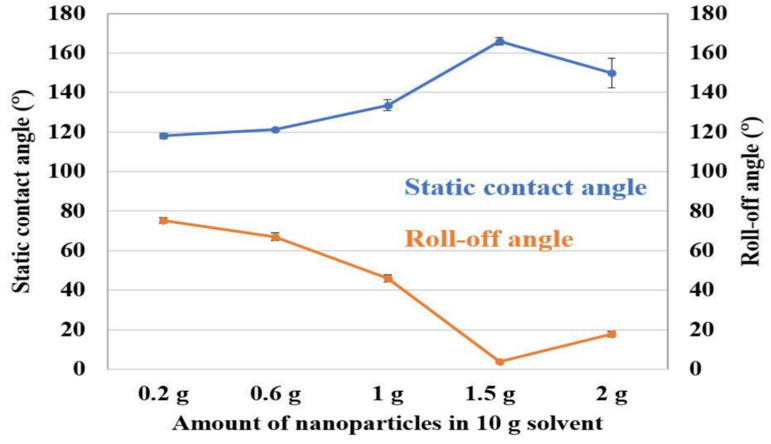
Static contact angle and roll off angle variation over different nanoparticle concentration. (Reprinted from Coatings 2018, 8(11), D. Sebastian, C. W. Yao, and I. Lian, Abrasion Resistance of Superhydrophobic Coatings on Aluminum Using PDMS/SiO_2_, 414, Copyright (2018) by the authors, Licensee MDPI, Basel, Switzerland. This article is an open access article distributed under the terms and conditions of the Creative Commons Attribution (CC BY) license (http://creativecommons.org/licenses/by/4.0/) (accessed on 23 November 2022)).

In surface engineering, wettability plays an important role. A material’s wettability indicates whether it is hydrophilic or hydrophobic. The degree of wetness that occurs between a solid and a liquid may be quantified by examining the wettability of the two phases. To fabricate an anticorrosive coating, the degree of wettability gives an assumption of the resistance to electrolyte solution. Structural properties, surface wettability, corrosion resistance and tribocorrosion behavior of boron and graphene oxide doped TiO_2_ nanotubes (TNT) were investigated by Acar et al. [30]. Contact angle measurements showed that the surface wettability of the synthesized nanotubes increased with increasing surface roughness and contact area, suggesting an increase in corrosion resistance. Mukherjee et al. [126] generated ripple-like micro-features with varying feature sizes by performing pulsed laser remelting of Ti6Al4V surfaces using a long-pulsed laser. The sessile drop method was used to calculate the surface contact angles. It was found that the droplets were more elongated after being exposed to laser surface remelting, indicating that the wettability of the surfaces was changed. Caha et al. [31] investigated the corrosion behavior of a titanium alloy with the inclusion of hard TiN particles. Ca and P were incorporated into the alloy and the composites by the MAO treatment, making them more biofunctional. Contact angle measurements were taken using a sessile drop technique with a 5 µL droplet of ultra-pure water to provide insight into the wettability of the surface. When compared to their untreated substrate, the contact angles of the bio-functionalized samples were much smaller and identical. The effect of nanoparticle concentration was investigated by Sebastian et al. [127]. For this purpose, varying SiO_2_ nanoparticles were introduced in a PDMS binder. Contact angle was measured by the sessile drop technique using 10 µL droplets. Figure 9 shows contact angle variation over varying nanoparticles. Contact angle increased as the nanoparticle content increased. After a 1.5 g nanoparticle concentration, contact angle dropped.

**Figure 10:**
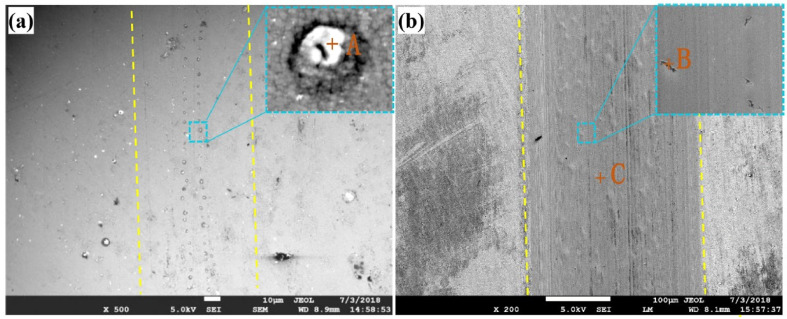
Wear morphology of (**a**) Cr-doped GLC coating, (**b**) Cu-doped GLC coating. +A, +B, and +C are locations for EDS analysis (Reprinted from Applied Materials and Interfaces, 10(42), X. Sui, R. Xu, J. Liu, S. Zhang, Y. Wu, J. Yang, and J. Hao, Tailoring the Tribocorrosion and Antifouling Performance of (Cr, Cu)-GLC Coatings for Marine Application, 36531–36539, Copyright (2018), with permission from American Chemical Society (ACS)).

Wear morphology characterization is another important method that is frequently used in tribocorrosion research to determine surface condition before and after wear. It elucidates the mechanism of wear before and after a tribocorrosion test. By using a multi-arc ion technique to deposit CrN and CrCN monolayers and CrN/CrCN multilayered micro/nano structured coatings on steel substrates, Wang et al. [32] were able to shed light on the tribocorrosion process of these materials. A field emission scanning electron microscope was used to examine wear morphology. The findings showed that the CrN/CrCN multilayer coating had a substantial amount of graphite-like phase and hard Cr-C compounds, both of which might be crucial in improving lubrication and hardness. Tribocorrosion characteristics of the Ti–25nb–3zr–2sn–3mo (TLM) alloy were shown to be enhanced by induction nitriding technology in simulated bodily fluids (SBF) by Dai et al. [128]. Tribocorrosion studies were conducted in SBF under varying electrochemical circumstances. An optical three-dimensional surface profilometer was used to examine the surface morphology after the tribocorrosion experiments. Wear morphology under OCP conditions confirmed that the nitrided sample’s abrasion marks were much narrower and shallower than those of the raw sample. Wear morphologies of the raw and nitrided samples under the cathodic potential under the same load were narrower and shallower compared to the wear morphology observed under an OCP, indicating that the application of a cathodic potential can effectively reduce the extent to which the titanium alloy wears. Corrosion at anodic potential generated a considerable increase in the degree of wear on the nitride sample compared to the breadth and depth of abrasion marks found for samples subjected to an OCP and cathodic potential. After conducting a systematic study of the effect of Cu and Cr doping on the microstructure, tribocorrosion, and antifouling properties of the coatings, Sui et al. [33] proposed a new concept for the preparation of multifunctional micro/nano-structured (Cr, Cu)-GLC coatings with excellent tribocorrosion and antifouling performance for marine applications. Figure 10a,b displays SEM images of the wear track to shed light on the coatings’ tribocorrosion behavior. Due to its significant hardness and low friction coefficient in saltwater, the Cr-doped coating showed almost no wear on its surface (Figure 10a). The coating’s wear track expanded noticeably once copper was doped into it (Figure 10b). The authors suggested groove and adhesive wear as the primary wear mechanisms of the Cu-doped coating.

## 3. Tribocorrosion Behavior of Nanocomposite/Nanoparticle Coatings

Inclusion of nanoparticles in the coating matrix has become a good practice to enhance corrosion resistance and resistance against plastic deformation and abrasion damage [69,71]. Inclusion of nanoparticles provides a distinctive micro/nano hierarchical structure that provides increased roughness to provide an air cushion. These air cushions trap air to form a passive air film that provides barrier protection against aggressive solutions [105]. Wang et al. [129] utilized a filtered cathodic vacuum arc at varying C_2_H_2_ flow rates to deposit nc-TiC/a-C:H nanocomposite coatings on Si wafer and AISI 304L stainless steel substrates. FESEM results revealed that nanocomposite coatings made from nc-TiC/a-C:H have a dense microstructure and a smooth morphology. To protect the coating from further influence of corrosion solutions, the periodic sliding test demonstrated that the passivation films of nc-TiC/a-C:H nanocomposite coatings had strong regeneration and repair ability on the sliding contact surface. The tribocorrosion behavior of low-friction Ti-Si-C-N nanocomposite coatings on ASTM F136 titanium alloy in phosphate-buffered solution was studied by Hatem et al. [130]. The samples were put through reciprocal sliding tribocorrosion testing in a PBS solution and compared to a bare Ti-6Al-4V alloy. Low-friction Ti-(Si)-C-N nanocomposite coatings showed a dramatic reduction in wear rate of at least 97% compared to the bare titanium alloy sample. Coating chemical composition and nanostructure were confirmed to be composed of Ti(C)N nanocrystallites embedded in an Si3N4 amorphous matrix within dispersed carbon amorphous sites, and this was shown to have a significant impact on the coated samples’ tribocorrosion performance. Fu et al. [131] conducted a comprehensive analysis of the microstructures, mechanical properties, and tribocorrosion performance of CrMoSiCN nanocomposite coatings prepared on Ti6Al4V via an unbalanced magnetron sputtering system. The results of the tribocorrosion tests demonstrated that the corrosion contribution to wear behaviors was the primary cause of failure in the Ti6Al4V alloy. Material loss is due to the synergistic effect of corrosion and wear behaviors, and the CrMoSiCN nanocomposite coating promoted pure wear behaviors throughout the tribocorrosion process. Cheng et al. [73] utilized an arc-spraying technique to create Al_2_O_3_/Al composite coatings with varying Al_2_O_3_ particle concentrations (0%, 10%, 20%, and 30% vol.%) in cored wire as feedstock. To simulate real-world conditions, tribocorrosion tests were conducted in a 3.5 wt.% NaCl solution while the coatings were subjected to potentiodynamic polarization. When compared to a pure Al coating, the coatings that had Al_2_O_3_ particles added showed significantly lower wear rates and coefficients of friction. The arc-sprayed Al-20%Al_2_O_3_ composite coating exhibited excellent tribocorrosion resistance. Wear patterns observed by scanning electron microscopy (SEM) indicate that delamination coupling of corrosion wear is the dominant tribocorrosion mechanism of composite coatings. Feng and Xiao [132] used laser cladding to create novel Ti-Al-(C, N) composite coatings for a TC4 substrate. A tribocorrosion test was performed using a reciprocating tribometer in simulated natural seawater. The findings demonstrated that the average friction coefficient was maintained at a low level by the presence of self-lubricating phases Ti2AlC and Ti2AlN in the composite coatings and by the generation of corrosion products with a certain lubrication effect during the friction process. The percentage of corrosion-induced wear increment in the synergistic effect increased from 61.92% to 71.08% as load increased, while the pure friction volume loss in TCN decreased from 89.89% to 87.83%. To create bio-functionalized surfaces incorporating ZrO_2_ nanoparticles, Costa et al. [55] coated Ti-40Nb alloy substrates with MAO at varying voltages. Tribocorrosion behavior, NP incorporation, and oxide growth mechanisms were discussed. ZrO_2_ nanoparticles (NPs) were found to improve the grown oxide coating’s tribological behavior, which could delay the brittleness and cracking typically seen in harder materials in response to intense mechanical loading. There has been a great deal of effort to mimic nature’s superhydrophobic qualities to discover new and exciting wetting properties along with superior anti-corrosion properties [133,134]. Superhydrophobic coatings are now used to provide barrier protection against corrosion damage in industrial structures, marine applications, automobiles, shipyards, and the oil and gas industries due to their superior water repellent nature and low-cost fabrication [135,136]. To increase the operational lifetime of a superhydrophobic coating, anti-wear properties can be measured along with corrosion properties by incorporating existing tribocorrosion experimental methods.

## 4. Conclusions

Since micro/nano structured coatings have great tribological, mechanical, and anticorrosion properties, the expanding field of surface coatings technology is moving in the direction of their widespread implementation across many industries. Coatings with nanoscale structures could be used in a wide range of technical fields, from maritime to aerospace to automotive to the medical field and beyond. Nanotechnology has allowed for the rapid development of protective coatings for surfaces with nanoparticles, allowing researchers to test out how well these coatings protect against chemical and physical wear. When wear and corrosion interact, tribocorrosion occurs. The relative motion of contacting metals in a corrosive environment is a common feature of engineering applications. To better understand the mechanisms of this complicated phenomenon, many studies have compared the tribocorrosion behavior of various micro/nano structured materials. When applied to systems where materials are unsuitable due to their higher costs and lower mechanical properties, knowledge of the tribocorrosion degradation mechanism of active metals and alloys may increase the useful life of these materials, with or without protective hierarchical structured coatings. Tribocorrosion studies face several obstacles, including the need to standardize test equipment, analysis methods, and testing procedures. Another purpose of this review, then, was to inform researchers and academics of recent advances in the field of tribocorrosion by providing a summary of the significant work done in this area.

This review discussed the basic approaches to tribocorrosion testing that have been used in recent studies of fabrication and characterization of micro/nano structured surfaces. It was noticed that the ball-and-pin-on-plate tribology test was the simplest and most effective method of simulating a wide variety of real-world systems. Most tribocorrosion studies combined potentiodynamic and potentiostatic polarization techniques to learn more about the passive film’s formation and its behavior in response to corrosion–wear simultaneous action. Measurements of open-circuit potential and electrochemical impedance spectroscopy were also used frequently. Alternative testing strategies that may help decode the intricate tribocorrosion phenomenon of the micro/nano structured coatings were also discussed. To better understand the micro and nanoscale behavior of corrosion products, micro/nanoindentation technology has proven to be an invaluable tool. Nano-DMA testing could be used to characterize material properties as a function of temperature, time, frequency, contact depth, or any combination of these parameters. The nano-DMA test can be used to surmise the extent of corrosion-induced damage. Coefficient of friction (COF) and scratch resistance behavior of micro/nano structured surfaces or coatings at micro-nano scales could be extracted using the dry sliding test in conjunction with the nano-scratch test. Friction mapping of micro/nano structured surfaces using lateral/friction force microscopy (LFM) is of growing interest in the scientific community.

Recent studies have revealed that coating systems based on nanocomposite materials offer a promising strategy for reducing the tribocorrosion phenomenon. The development of micro/nano structured superhydrophobic surfaces through nanocomposite or nanoparticle inclusion in the coating matrix has become a major focus of study in the field of anticorrosive coatings. There is still a need to investigate the wear properties of superhydrophobic coatings and their performance under synergistic wear–corrosion conditions, as this may reveal crucial characteristics to optimize the service life of the coatings under severe conditions.
